# Knowledge-driven enhancements for task composition in bioinformatics

**DOI:** 10.1186/1471-2105-10-S10-S12

**Published:** 2009-10-01

**Authors:** Karen Sutherland, Kenneth McLeod, Gus Ferguson, Albert Burger

**Affiliations:** 1 Department of Computer Science, Heriot-Watt University, Edinburgh, UK; 2 MRC Human Genetics Unit, Institute for Genetics and Molecular Medicine, Edinburgh, UK

**Keywords:** Graphical User Interface, Domain Model, Textual Representation, Argumentation System, Shopping Cart

## Abstract

**Background:**

A key application area of semantic technologies is the fast-developing field of bioinformatics. Sealife was a project within this field with the aim of creating semantics-based web browsing capabilities for the Life Sciences. This includes meaningfully linking significant terms from the text of a web page to executable web services. It also involves the semantic mark-up of biological terms, linking them to biomedical ontologies, then discovering and executing services based on terms that interest the user.

**Results:**

A system was produced which allows a user to identify terms of interest on a web page and subsequently connects these to a choice of web services which can make use of these inputs. Elements of Artificial Intelligence Planning build on this to present a choice of higher level goals, which can then be broken down to construct a workflow. An Argumentation System was implemented to evaluate the results produced by three different gene expression databases. An evaluation of these modules was carried out on users from a variety of backgrounds. Users with little knowledge of web services were able to achieve tasks that used several services in much less time than they would have taken to do this manually. The Argumentation System was also considered a useful resource and feedback was collected on the best way to present results.

**Conclusion:**

Overall the system represents a move forward in helping users to both construct workflows and analyse results by incorporating specific domain knowledge into the software. It also provides a mechanism by which web pages can be linked to web services. However, this work covers a specific domain and much co-ordinated effort is needed to make all web services available for use in such a way, i.e. the integration of underlying knowledge is a difficult but essential task.

## Background

Biologists have access to a wide range of web-based tools, and must generally use several to achieve their goals. Semantic Web technology has the potential to allow automation of the discovery and execution of web services, relieving biologists of the need to undertake repetitive, time-consuming and error-prone tasks, such as manual copying and pasting from one tool to another. So far the focus has been mainly on the bioinformatician creating complex workflows from a knowledge base of available web services, and not on the biologist who often lacks the necessary technical knowledge.

In addition, previous research has focused on the automation of workflow execution in bioinformatics without linking this to information normally browsed in web pages. For example, myExperiment [1] stores workflows created and executed within the Taverna Workbench [2], but there are no inherent links to resources users can browse. Other projects such as COHSE [3], focus on the semantic links between web pages.

The Sealife project [4] was conceived to tackle both these issues, i.e. linking browsable web pages to the corresponding web service infrastructure, and enabling a biologist to create workflows without being concerned with the underlying complexity.

### Sealife

**Figure 1:**
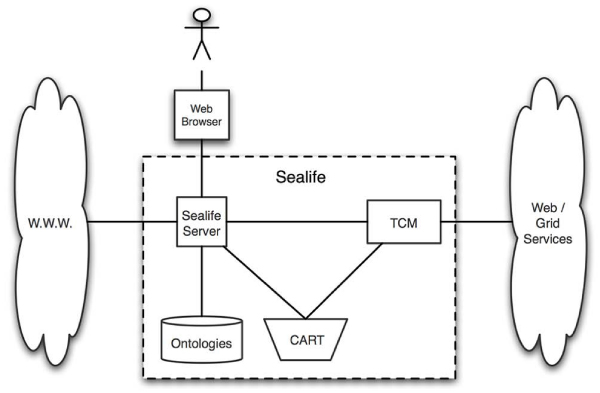
**Simplified architecture of Sealife**. A user browses a web page in which biological terms have been identified and annotated with semantic hyperlinks. When the user selects a link the Sealife server adds the associated term to the user's personal 'shopping cart' (CART). The Task Composition Manager (TCM) dynamically discovers services that require inputs of the same semantic type as those in the CART. The user can choose to execute any of these services through the TCM, and the result is presented by the browser.

Figure 1 shows the main components of the interactions within Sealife. A user browses a web page in which biological terms have been identified and annotated with semantic hyperlinks. These are created using text-mining techniques with a series of pre-existing ontologies [5]. When the user selects a link on the page, the Sealife server adds the associated term (with corresponding ontology ID) to the user's personal 'shopping cart' (CART). The Task Composition Manager (TCM) then dynamically discovers services that require inputs of the same semantic type as those in the CART. The user can choose to execute any of these services through the TCM, and the result is presented by the browser.

The text-mining components of the browser and their links to semantic information were investigated by the Sealife partners in Dresden and Sophia-Antipolis (e.g. [5–7]) and therefore will not be discussed here. The TCM (described in [8]) was created by the authors and provides the basic functionality for linking input terms found on a web page to relevant web services. Recent extensions to this module are described in this paper and aim to provide a richer, more useful environment for users. These extensions include the use of Artificial Intelligence (AI) techniques, such as Planning and Argumentation, which allow for the exploitation of domain knowledge, abstraction of technical detail and an in-depth analysis of results.

### Plan recognition and HTN planning

Within the life sciences, workflows are often used to answer a question or discover new knowledge. These questions can be viewed as high-level goals and the steps taken to achieve them form workflows. This process is identified in work by Hashmi *et al*. [9] and Tran *et al*. [10] which has shown that bioinformatics tasks can be abstracted out as an overall 'goal' to be accomplished and broken down into a series of sub-tasks in a hierarchy. In addition, this approach has the benefit of reflecting the way that people like to design, plan and solve problems [11]. It is for this reason that a hierarchical task network was chosen to model the information in this domain.

**Hierarchical Task Network (HTN) planning** [12] is an AI Planning technique used to break down a complex goal into executable steps. The domain is arranged as a hierarchy, placing abstract goals or tasks at the top of a hierarchical tree, and simple executable steps at the leaf nodes. There can be any number of intermediate nodes, which represent subgoals.

The Simple Hierarchical Ordered Planner (SHOP) [13] used in this work is an example of an HTN planner which orders actions in the way they are listed in the domain model. Previous work has included using the planner for web service composition [14] using OWL-S service descriptions, which were translated into the planner's own language. In bioinformatics most work describing web services has been done using templates from myGrid [15] and stored in the Biocatalogue repository [16].

**Plan recognition** introduces the possibility of determining what a user is interested in and may want to do. In traditional plan recognition a sequence of steps performed by an intelligent agent (e.g. a human) is observed and used to determine the goal of that agent [17]. Within Sealife, the 'steps' that are performed are the addition of semantically meaningful terms to the CART (e.g. "mouse heart structure"). In order to link these terms to web services, the semantic type of the term is determined (in this case "mouse anatomy ID") and all available web services are searched to identify those which take this type of input. The way this is done is described in the Methods section.

In this work the system that makes use of these planning techniques is called the Goal Generation And Planning System (GGAPS). Although it is used in the context of Sealife with terms retrieved from a web page it can also function independently.

These aspects of AI Planning were introduced to help life scientists to navigate the growing amount of online tools available, and enable them to construct and manage workflows for themselves. Other work has attempted this in different ways. For example, the Bio-jETI [18] project makes use of model checking techniques to allow users to connect web services in a way that does not require extensive technical knowledge. The workflows are constructed in a step-by-step fashion, which is in contrast to the abstraction process used in the current paper.

### Argumentation module

Due to the complexities of the biological domain, and differing interpretations of data, experimental results vary, leading to variations and contradictions in online data resources. Inconsistencies can arise both within and between online resources which require reconciliation by researchers.

**Argumentation theory** is used to allow computers to argue, or to help humans argue [19]. One approach is to perform reasoning over inconsistent information with arguments generated for and against a statement being true. These arguments can then be used by the system, or human user, to make a decision on the validity of the statement. Argumentation can be modelled as a dialogue between two people [20], mimicking a natural process that is intuitive to human users [21].

Argumentation Schemes [22] provide a template for an argument in natural language and comprise an inference rule and a set of critical questions. The rule forms an argument when it is instantiated and the questions are used as a heuristic for the evaluation of such an argument. The transformation from natural language schemes to formal logic inference rules, required by many systems that perform Argumentation, was documented by Verheij [23].

Argumentation has been applied to medicine [24], law [25] and practical reasoning [26]. Medical uses of Argumentation vary from decision support systems in clinical practice [27, 28] to systems generating explanations of diagnosis for patients [21]. Little work has been done using Argumentation in bioinformatics, apart from its use to evaluate the output of a protein prediction tool [29].

Argumentation is used in Sealife to resolve inconsistencies across biological data resources by creating arguments for and against potential answers to a query [30]. Such arguments are presented to the user, helping them to identify the most credible results [31].

Existing mechanisms to integrate data and resolve inconsistencies include turning multiple possible values into a single value (Data Fusion [32]), and selecting the best of multiple query plans for the resources according to information quality criteria [33]. Sealife does not aim to automatically resolve conflicts, but assists biologists in resolving the differences themselves.

### Domain

The work in this paper is carried out within the context of the gene expression domain, based on the practice of using the mouse embryo as a model for early human development.

### This paper

The remainder of this paper outlines the design and implementation of each of these modules and how they were evaluated. The results of the evaluation are given, followed by a discussion of the difficulties encountered, a comparison with other systems, and the possibilities for future work. The conclusion summarises the paper.

## Methods

The methods cover four main areas: the design and implementation of the basic TCM module; the implementation of GGAPS; the implementation of the Argumentation System; and the evaluation of these systems. Table 1 outlines the main components of these modules.

**Table 1:** System components showing elements re-used from other projects.

Sealife component	Re-used from other projects
TCM	Feta [34] search engine
GGAPS	SHOP [13] HTN planner
Argumentation System	ASPIC [43] argumentation engine
Workflow Execution	myGrid [15] workflow execution engine and language (Scufl)

### Task composition manager (TCM)

The TCM was developed using various myGrid [15] components, including: a service discovery engine (Feta [34]), a workflow enactment engine, and the workflow execution language (Scufl[35]). In order for Feta to find services, they had to be described using a proprietary XML template to produce a semantic description. The myGrid domain ontology [34] was used for these descriptions and extended for the purposes of the gene expression use case. Terms from the ontology are classified semantically and used to search for services which take inputs of this semantic type.

For services to be executed, a workflow must be written in Scufl. This can be manufactured using the Taverna Workbench or re-used from a repository such as myExperiment. For the purposes of the TCM, Scufl workflows were created by hand and stored locally. A semantic description for each workflow was produced so that Feta could return workflows as well as services in response to a query.

The implementation of the TCM and the issues encountered are described in detail in [8].

### Goal generation and planning system (GGAPS)

GGAPS includes both the HTN Planner (SHOP) and the plan recognition module. Both use the same hierarchical domain model, which was limited to a small part of the gene expression domain in order to demonstrate the principle and minimise difficulties with web service availability.

#### Use case

The use case involves the discovery of genes that may play a part in the development of a particular human structure (e.g. heart or brain). This requires finding the equivalent structure in the mouse embryo, determining the genes expressed in this structure during development, and then relating them back to the equivalent human genes.

**Figure 2:**
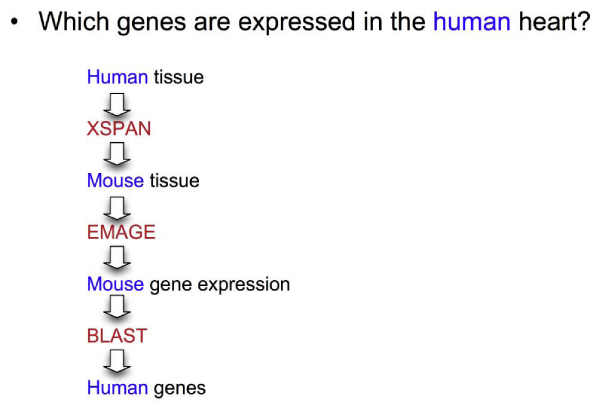
**Simple workflow**. Simplified version of the process used to find out which genes are involved in the development of a particular human structure. First the equivalent structure is identified in the mouse embryo and the genes expressed here are found. These genes are 'BLASTed' to find homologous human genes. XSPAN is a cross-species anatomy database, and EMAGE is a mouse gene expression database.

Figure 2 shows the outline of a workflow connecting these services. Either manual cutting and pasting is required to operate each service via an online user interface, or the web services themselves must be connected computationally. Usually a life scientist with little technical knowledge of web services would proceed in a step-by-step fashion online.

**Figure 3:**
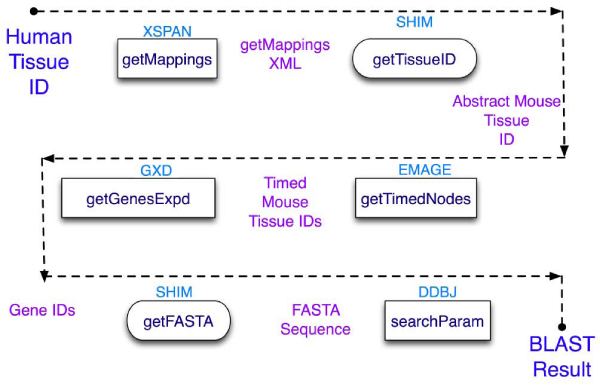
**Detailed workflow**. This workflow shows the web services involved in mapping the human structure onto a mouse structure, finding the mouse genes expressed in this structure, and finally discovering the corresponding human genes. Shims are used to convert the output of one service into a format suitable for the next service.

Figure 3 shows how this workflow can be implemented using web services connected computationally. First the equivalent structure in the mouse is identified using XSPAN [36], a database of links between anatomy ontologies. The mouse structure is then used to query a mouse gene expression database (GXD [37]) with the *getTimedNodes* operation converting the abstract concept of the mouse structure to a specific developmental stage. The mouse genes identified are then compared to human genes using BLAST [38] by searching biological databases for homologous sequences.

Mismatch of output and input types from one service to the next requires use of a shim [39], a local service that converts the output of one service into the format required by the following service e.g. the *getMappings* operation in Figure 3 produces an XML file, but the subsequent service requires an Anatomy ID (which is obtained from the XML file). Steps corresponding to shims are included in the domain model.

#### Creating the domain model

**Figure 4:**
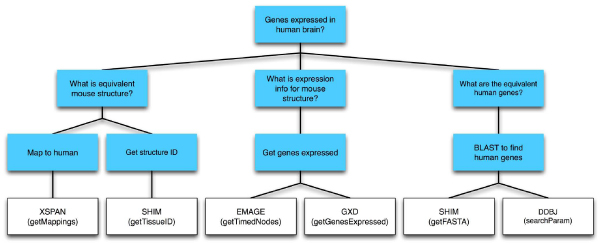
**HTN domain model**. The domain was modelled in the form of a hierarchical task network where an abstract goal is broken down into a number of executable steps. The leaf nodes correspond to individual executable web services.

An HTN domain model was created to model the information contained in the workflow and implicit in the researcher's query. At the most abstract level the goal was formulated as a question, i.e. *Which genes are expressed in the human brain?* and placed at the root of the graph (Figure 4). The question was then broken down into the sub-goals and executable steps needed to be performed to answer it. In the hierarchical task network steps representing individual web services are placed at the leaf nodes and intermediate steps representing sub-goals link these nodes. These sub-goals can also be expressed as questions.

Individual web services could potentially be combined in an almost infinite number of ways, but only combinations that make both biological and technical sense were expressed in the domain model. In addition, many web services are not semantically marked up and this needs to be done before they can be fully exploited.

### Using the model to find goals

The plan recognition element of GGAPS uses an algorithm that simply traverses the domain model searching for goals or tasks that take an input of the same semantic type as the input item in the cart (or supplied by a user). At present only one input term at a time has been used for testing purposes.

**Figure 5:**
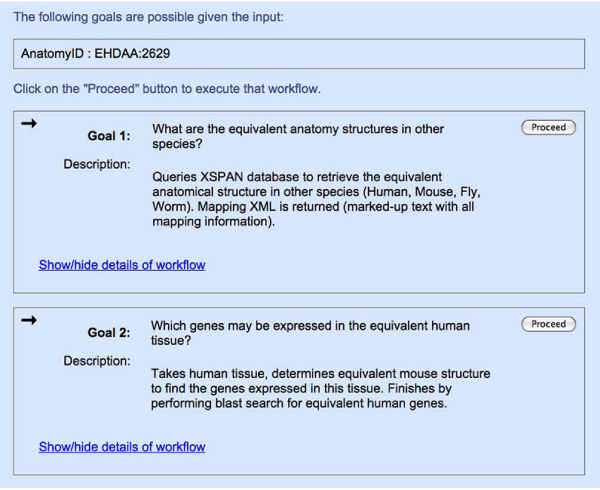
**Screenshot showing a choice of goals**. GGAPS screenshot capturing two possible goals and their descriptions.

The domain model is translated into Java using the concept of a 'Task' class which allows storage of information related to particular tasks (or goals) in Task objects. A list of goals is produced from the domain model and associated with information on its parents and children, allowing the user to be informed of how this goal would potentially be achieved. A natural language description of the goal is also presented to the user to help them to decide whether or not it is of interest (Figure 5). Once the goal has been selected, the user can choose to execute it.

### Using the model to provide a plan of execution

The SHOP planner is used to produce a plan of execution from the domain model and the steps converted into a workflow (as shown in Figure 3). The domain model is first translated into the Lisp-like format used by the planner, and the problem must be modelled in the same way. As the planner operates in the order in which the tasks should be executed, this attribute was exploited in automatically creating the Scufl file. However there were some limitations on the amount of information that could be passed from the domain model to the final plan.

An alternative method was to use the same Task objects used in goal discovery to retrieve information on children and/or parents of goals or tasks. The domain model can be searched for the relevant goal and then information about its children is used to break down the goal into its constituent steps. The names of the operations which need to be run and the location of the WSDL are contained in the leaf nodes, which is helpful for the automatic creation of the Scufl file. However this method has the disadvantage of not being as sophisticated as the SHOP planner which is a sound and complete purpose-built HTN planner.

The information obtained from the domain model through either approach is used to automatically create a Scufl file which is executed by the myGrid workflow enactment engine. However, any valid workflow execution language and enactment engine could be used. The resultant Scufl file contains information describing the location of web services and how data should be linked between them.

**Figure 6:**
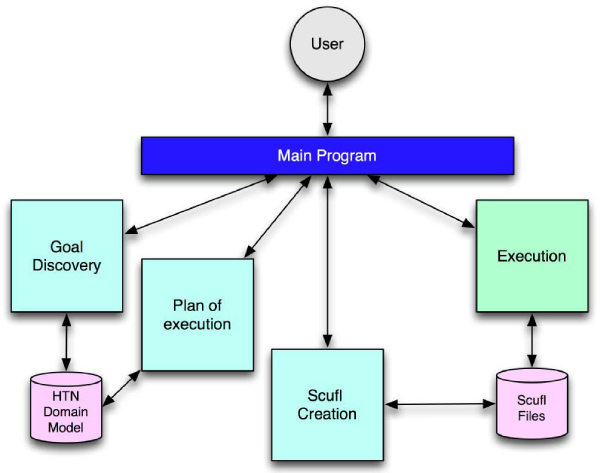
**GGAPS system overview**. Diagram showing subsequent steps of goal discovery process. After plan recognition a set of goals is presented the user. They choose one and a plan of execution is formed. A Scufl workflow is produced from this plan and subsequently executed using a workflow enactment engine.

Figure 6 summarises all the processes involved in taking an input from the user, offering goals, producing a plan and finally executing a workflow. This could replace the original TCM which simply returned a list of single services or pre-canned workflows.

### Argumentation

The use case for the argumentation module featured experimental results from three databases which publish gene expression information for the Developmental Mouse: EMAGE [40], GXD [37] and CGAP [41].

EMAGE and GXD publish *in situ* gene expression information using the EMAP anatomy ontology [42], but differences exist as some experiments published in EMAGE are not available in GXD and vice versa. Furthermore GXD contains only results mapped to the EMAP anatomy ontology. EMAGE distinguishes between these (Textual Annotations) and results are mapped to a 4D model of the organism (Spatial Annotations).

CGAP's mouse data (from SAGE experiments) is not tied to the EMAP anatomy ontology, but to their own anatomy ontology. There is no direct one-to-one mapping between these ontologies, therefore only data subsets corresponding to individual expert-created mappings between the ontologies can be used.

The argumentation engine used for Sealife was created as part of the ASPIC project [43] and takes domain information as well as a series of inference rules to create arguments by backward chaining through the rules in response to a query from the user.

Expert knowledge provided by a curator of the EMAGE database was formalised using argumentation schemes and coded as the first order predicate logic rules required by ASPIC (the translation based on the work of Verheij).

Two types of schemes were created, the first relating to the user's trust in resources (e.g. EMAGE), journals, individual researchers, and techniques (e.g. Spatial Annotations), and the second for broadly accepted inferences (e.g. Textual Annotations are generally more reliable than Spatial Annotations).

With the aid of the expert, the schemes were ranked and scored for importance, and the scores associated with ASPIC's rules. Scores are used by the engine to determine the strength of arguments and thus resolve conflict between them – the strongest argument wins any conflict.

Clients were developed to use online database interfaces (initially only EMAGE [44] and GXD [45]) to obtain data and convert it for use with ASPIC using Verheij's translation technique. Users' trust levels in relevant researchers and journals were ascertained and presented to ASPIC.

**Figure 7:**
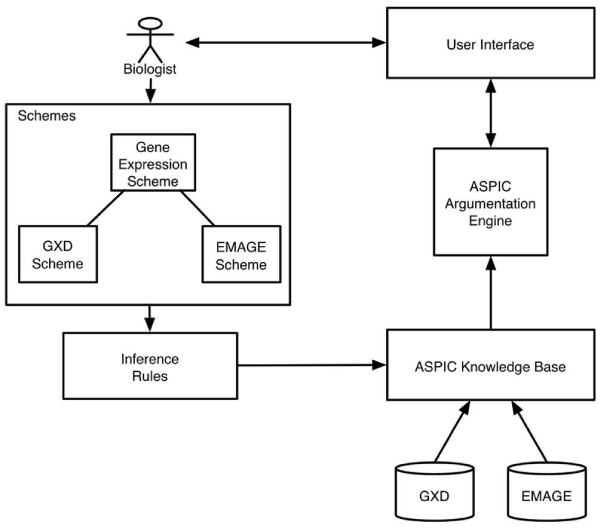
**Simplified architecture of argumentation system**. A human expert created Argumentation Schemes that modelled their knowledge of how to interpret the gene expression results in EMAGE and GXD (two *in-situ* gene expression databases for the Developmental Mouse). These schemes were converted into rules, and used by the ASPIC Argumentation engine to create arguments. The other ingredient for arguments, is experimental information from EMAGE and GXD – this was pulled when the user specified a gene-structure pair through the User Interface. Arguments were created in response to a query – is *gene* expressed in *structure?* – and returned to the user.

The domain data and expert knowledge in ASPIC's knowledge base can be queried (*Is the gene expressed in the structure?*) and the resulting arguments are displayed to the user. A simplified architecture of the system can be seen in Figure 7.

Using a text based user interface, an initial expert evaluation of the system was conducted with the development team, including the biological expert. This evaluation enabled fine-tuning of the type and content of the arguments generated by the system and identified two main issues: (*i*) the need for a graphical user interface (GUI), and (*ii*) the need to include additional databases to EMAGE and GXD. Further details on the above can be found in [31].

**Figure 8:**
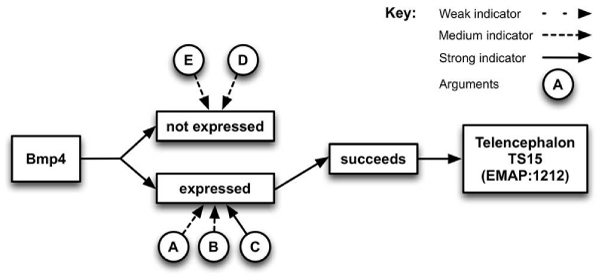
**Visual summary of arguments produced by argumentation system**. An example summary presenting the arguments for the query: *is the gene Bmp4 expressed in the Telencephalon in TS15?* Different types of arrows are used to indicate the strengths of the arguments, and the conclusion – determined by the strongest argument – is indicated by the complete path to the *succeeds* box. The summary shows that *Bmp4* is expressed in the Telencephalon in TS15 (which has EMAP anatomy ontology ID *EMAP:1212*).

As a result, CGAP was included in the system and a prototype GUI designed and implemented. Requirements analysis indicated expert users wanted a textual representation of the whole argument and a visual summary of the arguments' relationships – showing those for and those against the gene being expressed. An example summary presenting the arguments for the query: *Is the gene Bmp4 expressed in the Telencephalon in TS15?* can be seen in Figure 8. Different types of arrows are used to indicate the strengths of the arguments, and the conclusion – determined by the strongest argument – is indicated by the complete path to the *succeeds* box. For example, in Figure 8 the summary shows that *Bmp4* is expressed in the Telencephalon in TS15 (which has EMAP ontology ID *EMAP:1212*).

This use of both visual and textual representations of the arguments was the focus of the Argumentation System evaluation discussed below.

### Evaluation

The aims of the evaluation were to: design and implement a GUI; determine the usability and functionality of GGAPS and the Argumentation System; evaluate different data representations for the display of results; and evaluate the Argumentation methodology.

A prototype GUI was designed by the development team and piloted internally. The GUI requirements included that it be simple, include a shopping cart, and have a common appearance and functionality for both GGAPS and the Argumentation System. The prototype was implemented in HTML and Javascript so that it could run in any standard web browser.

#### Evaluation structure

Scenarios for evaluation of GGAPS and the Argumentation System were developed based on the relevant use cases and tested internally using structured walkthroughs and expert evaluation. To avoid variations in the availability and speed of third-party online resources, data required for the scenarios was generated using the systems, and hard-coded into the GUI with appropriate delays to represent online resource access.

User evaluations were structured using a script, one observer to interact with the user and another to record timings, errors, user comments and interactions. Final protocols were piloted with postgraduate Computer Science students.

The evaluations were timed, with splits to enable any online resource access to be allowed for in the analysis. Users were encouraged to comment freely and ask questions or for help at any stage.

General usability questions were adapted from Shneiderman's Questionnaire for User Interaction Satisfaction (QUIS) [46] and the same format was used where appropriate for the other questionnaires.

#### User group

Eighteen users were recruited, ten from the Edinburgh Mouse Atlas Project (EMAP) [47] (in various roles from system developers to biological database curators), and eight Computer or Life Sciences students at Heriot-Watt University. For analysis, users were divided into two groups: biologists and non-biologists (nine in each), based on background and experience. The biologists included gene expression experts, who gave additional expert opinion on some aspects of the system.

#### Scenarios

The first GGAPS scenario (referred to as the "Manual Scenario") required users to determine which genes might be expressed in the developing human brain by accessing typical online bioinformatics resources through standard browser-based interfaces. Users were required to access the XSPAN Cross-species Anatomy Database, EMAGE Mouse Atlas, GXD and Uniprot [48] sites, as well as perform a BLAST search and record a detail from the result.

The second GGAPS scenario required the use of the GGAPS GUI to generate possible goals from a term selected from the CART. After selecting the appropriate goal to determine which genes might be expressed in the developing human brain, the user ran the workflow using GGAPS and recorded a detail from the result.

The Argumentation scenarios required users to select a resource from the CART (a structure and gene in the developing mouse brain) and use the Argumentation GUI to generate arguments for and against the gene being expressed in the structure. For the first scenario default values for database, journal and researcher trust were used, and for the second scenario, the user set parameters to specific values.

#### Questionnaires

After each scenario, users completed a questionnaire on the aspects of the system covered by the scenario. Where appropriate, questions used a 9 point Likert scale [49] to enable users to rate usability and functionality. A number of questions were focused on methods of representing the results in both systems, particularly the Argumentation System.

**Figure 9:**
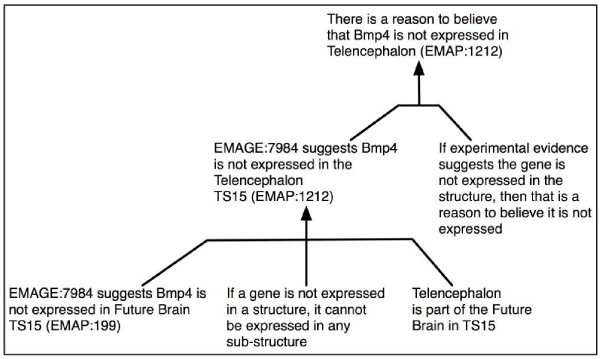
**A possible graphical representation of an argument**. A typical argument from the GUI interface of the Argumentation System presented as a graph, with the component parts and the relationships between them identified. This argument uses the fact that if a gene is not expressed in a structure, it cannot be expressed in a sub-component of that structure to suggest that *Bmp4* is not expressed in the Future Brain in Theiler Stage 15 of the Developmental Mouse.

Argumentation results were presented in the form of: a summary, a graphical representation and a textual representation and users indicated which elements they used to reach conclusions on the individual arguments generated by the system. They were also asked to draw conclusions and comment on different forms of graphical representation, for example a graphical representation of an argument (e.g. Figure 9), and the visual summary of arguments (e.g. Figure 8).

After completing all scenarios, users were asked to rate the usability of the system as a whole.

## Results

The results of this paper are again split into multiple categories. Firstly there are implemented prototypes, for the TCM, GGAPS and Argumentation System, then there are the results of the evaluation. The evaluation contains work on Argumentation, GGAPS, and the shared GUI of these systems.

### TCM prototype

A prototype of the TCM's core functionality (service discovery and enactment) currently exists. It offers a simple command line interface that allows users to search for, then execute, services in a limited domain. Currently this domain includes the human and developmental mouse anatomies, *in-situ* gene expression for the developmental mouse, and a small set of related tools such as BLAST. Though the prototype is simple, it conveys the power of this technology if one is willing to invest in the linking of the underlying resources.

### GGAPS prototype

The basic GGAPS prototype consists of a simple command line interface that guides the user through the system from choosing a goal to executing the plan. This interface is not linked to the CART so for testing purposes a type of input is chosen (e.g. a mouse structure) from a short list, and the system produces a list of goals relating to this input. Once a goal has been chosen the plan of execution is displayed and, if desired, subsequently executed.

The online prototype enabled connection to the CART and allowed more detailed representation of the plan for the user. Three main screens were used for the processes of (1) selecting an item from the cart, (2) viewing the goals produced and (3) displaying the results. The goals were presented as natural language descriptions but also allowed the users to click on a link to show more details such as the inputs required and the outputs produced.

### Argumentation prototypes

Early versions of the Argumentation System used a simple text-based interface which displayed the argumentation results as text. The display was based on ASPIC's internal representation of the arguments, but expert users indicated these representations were neither intuitive nor attractive.

**Figure 10:**
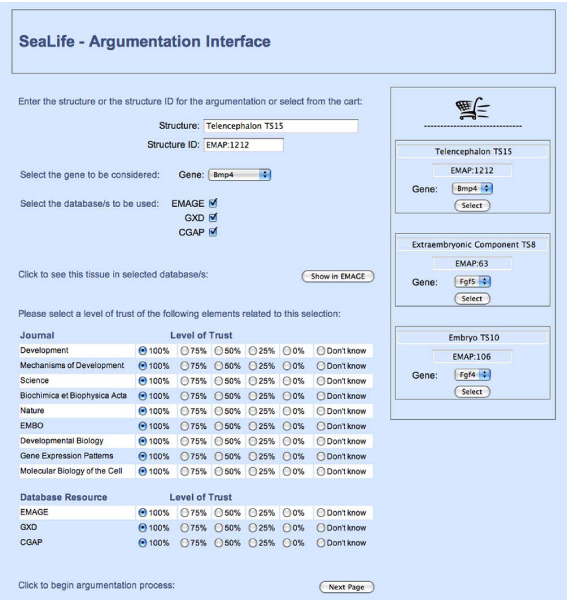
**Start page for argumentation system GUI**. The initial page for the Graphical User Interface of the Argumentation System asks the user to select a gene-structure pair they wish to investigate, and then asks for their level of trust regarding the resources (EMAGE, GXD, and CGAP) and relevant journals.

**Figure 11:**
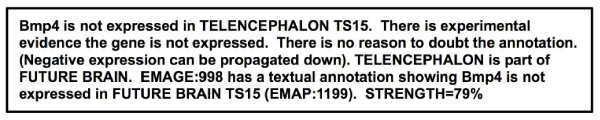
**Textual representation of an argument**. A typical argument from the improved GUI interface of the Argumentation System. This argument uses the fact that if a gene is not expressed in a structure, it cannot be expressed in a sub-component of that structure to suggest that *Bmp4* is not expressed in the Future Brain in Theiler Stage 15 of the Developmental Mouse. This is a textual representation of the argument presented graphically in Figure 9.

These issues were addressed during the development of the GUI. It allows users to specify a gene and structure, then select trust levels for resources, journals, researchers and the relevant experiments before proceeding with the argumentation process (see Figure 10). The results consist of a brief summary statement, the summary image (e.g. Figure 8) and an evolved textual representation of the arguments (e.g. Figure 11).

The evolved representation of an argument was developed in response to the requirement of the expert biologists for arguments in natural language. The results of the evaluation of these representation methods are detailed below.

### Argumentation and GGAPS evaluation results

The evaluation results in this section are presented in greater detail in [50].

A combined GUI for GGAPS and the Argumentation System was necessary to enable evaluation of the usability, functionality and display of results generated by the systems. User comments were positive with regard to general simplicity of design and layout of the GUI, but as the CART was loaded with pre-selected resources, users had some difficulty in understanding how the concept was used in the system and the scenarios did not address how these had been obtained.

As part of determining their background and experience, users rated their familiarity with six bioinformatics tools and databases used in the evaluation. The non-biologists were generally unfamiliar with any, while familiarity amongst the biologists varied considerably, with some using most of the tools continually, and others just a few occasionally.

The mean duration for the entire evaluation (from the start of the GGAPS section to the end of the Argumentation section) was 31 minutes 8 seconds (range 20 m 25 sec – 46 m 35 secs, Std. Dev. 7 m 21 secs). There were no significant differences between times taken by the biologist and non-biologist groups. In addition, there was no significant difference in the times taken by the different groups for either the manual or GGAPS scenarios or in the times taken by the two groups to complete the Argumentation System scenarios.

The modified Shneiderman questionnaire used to evaluate the overall usability of the Sealife system (both GGAPS and the Argumentation System) collected user ratings in the following areas: user reactions to system overall, screen sequence and layout, use of terminology, system information and error handling, and system capability. The responses were consistent, with the majority of responses positive – 73.7% greater than 5 on a scale of 1 (Bad) to 9 (Good). There were no significant differences between the ratings of the non-biologist and biologist groups.

**Figure 12:**
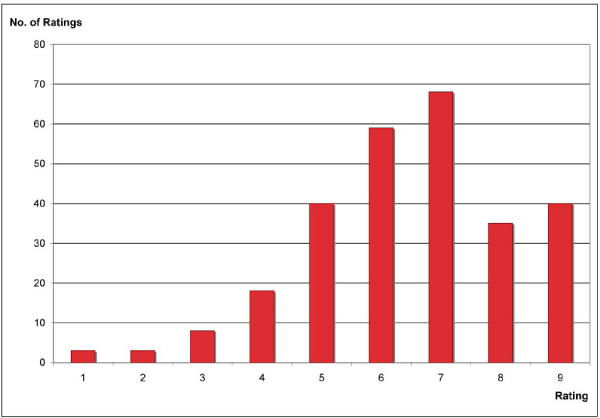
**Sealife usability**. An overall impression of the distribution of user ratings for usability of the SEALIFE system is shown by adding all the scores across the seventeen questions on general system usability adapted from the QUIS.

A graphical representation comparing the overall usability of the Sealife system is shown in Figure 12. The ratings given by each user for each question were added to give an overall impression of the distribution of ratings for the system.

### GGAPS evaluation

**Figure 13:**
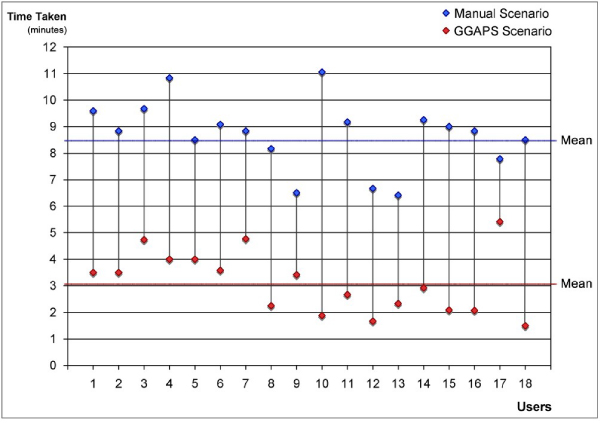
**Manual vs. GGAPS: time to complete scenarios**. Illustrates the range of differences between the times taken by users to complete the manual scenario and the GGAPS scenario.

There was a significant difference in the times users took to complete the manual scenario compared with the GGAPS scenario (p = 0.000), with a mean time for the manual scenario of 8 minutes 42 seconds (range 6 m 25 s – 11 m 17 s) and for the GGAPS scenario 3 minutes 8 seconds (range 1 m 30 s – 5 m 25 s). These differences are illustrated in Figure 13 and show the relevant efficiency of the automated system compared with the manual scenario.

At the end of the two scenarios users compared the usability of the manual system and the automated GGAPS system in terms of their overall impressions. Rating the aspects *Difficult...Easy*, *Time-consuming...Quick*, *Inefficient...Efficient*, and *Hard to understand...Easily understood*, on scales of 1 to 9, the medians of the ratings for the manual system ranged from 4 to 5, while the medians of those for the GGAPS system ranged from 7 to 8. Taking all users, the differences between the ratings of the manual system and GGAPS on each question were significant (all scores p ≤ 0.002). There were no significant differences between the ratings of the non-biologist and biologist groups within the users.

**Figure 14:**
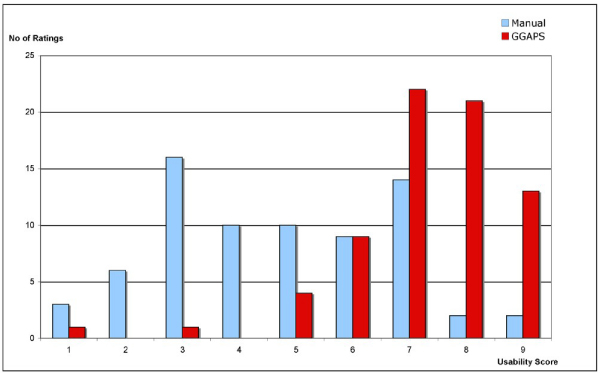
**GGAPS usability**. Shows the distribution of the usability ratings for the manual and GGAPS systems by adding the ratings across the four questions addressing the usability of each system.

A graphical representation comparing the overall usability for the manual and GGAPS scenarios is shown in Figure 14. The ratings given by each user for each question were added to give an overall impression of the distribution of ratings for each scenario. The results show that GGAPS generally scored higher on ratings of usability than the manual system.

To direct questions on the amount of control and transparency of the GGAPS system, responses indicated that although the manual system offered more control of the process than the GGAPS system, the latter was easier to use without instructions. Users also felt the way information was presented in the GGAPS system was better than in the manual system. Very few users (11%) actively sought further information by using the available links to view more details of the goals, and only 4 (22%) used the link to view the full BLAST report at the end of the scenario.

Users liked the presentation of the tasks as goals, as well as the choice and breakdown of goals. Four users (22%) did not like the presentation of the results and 2 (11%) did not like the CART.

Overall, the results indicate that GGAPS is relatively easy to use and would save time for the user.

### Argumentation system evaluation

In both scenarios the responses of most of the non-biologist group showed correct understanding of the process and results, which contrasted with the biologist group. The responses of more than half (56%) of the latter group indicated that they had used their own knowledge of the data to over-ride the Argumentation System results, particularly in the second scenario. Comments showed that the expert biologists did not accept the arbitrarily imposed changes in the trust status of the second evaluation scenario.

The biologist group scored their understanding of the arguments significantly higher (median 7) than the non-biologists (median 3) on a scale of 1 to 9 (*Not understood at all – Completely understood*) (p = 0.0121). The low levels of understanding among the non-biologists appeared to be largely due to lack of background biological knowledge.

Fifteen users (83%) rated the amount of information presented between 4 and 6 (1 – *Too little* to 9 – *Too much*) with 12 (67%) scoring it at 5 (just right). This was felt to indicate that the presentation of the results from the Argumentation System is clear and allows non-biologist users to reach the correct conclusion, while biologists have issues with trusting the system and its results.

When asked to compare the graphical representation of an argument (e.g. Figure 9) to a textual representation (e.g. Figure 11), nine users (50%) preferred the graphical and 8 (44%) the textual, with 1 (6%) undecided. A number of users felt the graphical representation could be improved by having the decision tree run from top-down, rather than bottom-up and that both graphical and textual representations should contain more in-depth explanation of the argument.

In the Argumentation scenarios, the results page was headed by a single line summary representing the conclusion the system had drawn, followed by the summary diagram, and then the textual representation of all the arguments. Most users (with the exception of two outliers) found the summary diagram very easy to understand, with the strength of argument correctly identified in 47 out of 54 instances (87%).

Six users (33%) employed all three elements (of the results page) to reach their decision on whether the gene was expressed or not. Three users (16%) made use of only the diagram, three only the text and the rest used combinations of two elements, with no particular combination preferred. This indicates the need for all three representations to be presented by the system.

Valuable feedback was obtained from user comments and answers to open-ended questions eliciting opinions on aspects of the system. These included suggestions regarding additional online resources, and inclusion of a tool-tip style help and a tutorial. These results will feed back into development of the system.

## Discussion

The life sciences domain is a highly specialised area of science which ranges from sophisticated high-technology services that support research and clinical services, through to individual interpersonal interactions, such as the clinical consultation. There is a correspondingly wide range of people working in the area, many with extremely detailed and in-depth knowledge of their specialist fields. These factors pose significant challenges for systems developers within these domains, particularly when systems attempt to bridge across specialities and enable users to access and use data, services and expert knowledge from outwith their own speciality.

It has become obvious that significant efforts are necessary to capture all the relevant semantics of the domain. This not only includes the domain ontologies, such as mouse and human anatomy for our gene expression use cases, but also ontologies to semantically describe services, capture the domain model used by GGAPS, and represent the rules that govern the argumentation mechanism across resources. Not only are these required in isolation, they must also be consistent between each other. For example, the concept of an anatomical structure in the mouse anatomy ontology needs to be consistent with the same concept in the service descriptions, the planning domain model and the argumentation representations.

Once the background systems have been implemented, evaluation generally requires some form of user interface. The gathering of the requirements for the user interface, designing and implementing it, and then evaluating both the interface and the underlying system requires considerable time and expertise. At the same time, the outcomes from these evaluations typically require iterations of changes that reach deep into the underlying systems and services.

All this development and evaluation takes place above the level of the basic resources used by the systems being developed. For the most part these resources have been developed and run by third parties and therefore many of the inherent limitations and restraints cannot be addressed or influenced directly by the top-level systems developers, but must instead be worked around.

The number of challenges and difficulties faced, by developers of systems that aim to bring together and use various web-based third-party resources, increases exponentially with each technology and layer of functionality added to the system.

## Conclusion

The primary aim of this work was to provide a module within Sealife that would enable the semantic linking of web pages to web services. In order to achieve this goal, tools originating from the myGrid project were used, connected together and added to novel ideas and code. The outcome was a software module (the TCM) that semantically discovers web service operations based on items the user places in a shopping cart.

To extend this further, GGAPS was developed to allow a user to be able to work at a more abstract level relevant to the questions asked in their research. Instead of having to plug together web services at a technical level, as in other workflow creation tools, the user is instead offered a choice of higher level goals. These are then broken down into subgoals by an HTN Planner and subsequently executed. The Argumentation System presents a further level of functionality to the user, allowing them to critically evaluate the results that have been returned.

These systems demonstrate that it is possible to integrate web pages with web services in a dynamic approach that exploits the semantics of the domain, and which requires little technical understanding of the computational elements by the end user. The work described in this paper also illustrates that time, effort and ingenuity are required to develop systems that will provide users with the functionality of a number of web-based services in a single usable system. The complexity of such a development is multiplied each time a disparate service is added and increased again when additional technologies are employed. A whole new set of technologies and skills are therefore required to capture and model expert domain knowledge in order to maximise the potential of the Semantic Web.
